# The cost-effectiveness of increasing alcohol taxes: a modelling study

**DOI:** 10.1186/1741-7015-6-36

**Published:** 2008-11-28

**Authors:** Matthijs van den Berg, Pieter HM van Baal, Luqman Tariq, Albertine J Schuit, G Ardine de Wit, Rudolf T Hoogenveen

**Affiliations:** 1 Centre for Public Health Forecasting, National Institute of Public Health and the Environment, PO Box 1, 3720 BA Bilthoven, The Netherlands; 2 Centre for Prevention and Health Services Research, National Institute of Public Health and the Environment, PO Box 1, 3720 BA Bilthoven, The Netherlands

## Abstract

**Background:**

Excessive alcohol use increases risks of chronic diseases such as coronary heart disease and several types of cancer, with associated losses of quality of life and life-years. Alcohol taxes can be considered as a public health instrument as they are known to be able to decrease alcohol consumption. In this paper, we estimate the cost-effectiveness of an alcohol tax increase for the entire Dutch population from a health-care perspective focusing on health benefits and health-care costs in alcohol users.

**Methods:**

The chronic disease model of the National Institute for Public Health and the Environment was used to extrapolate from decreased alcohol consumption due to tax increases to effects on health-care costs, life-years gained and quality-adjusted life-years gained, A Dutch scenario in which tax increases for beer are planned, and a Swedish scenario representing one of the highest alcohol taxes in Europe, were compared with current practice in the Netherlands. To estimate cost-effectiveness ratios, yearly differences in model outcomes between intervention and current practice scenarios were discounted and added over the time horizon of 100 years to find net present values for incremental life-years gained, quality-adjusted life-years gained, and health-care costs.

**Results:**

In the Swedish scenario, many more quality-adjusted life-years were gained than in the Dutch scenario, but both scenarios had almost equal incremental cost-effectiveness ratios: €5100 per quality-adjusted life-year and €5300 per quality-adjusted life-year, respectively.

**Conclusion:**

Focusing on health-care costs and health consequences for drinkers, an alcohol tax increase is a cost-effective policy instrument.

## Background

Excessive alcohol use is a cause of morbidity and mortality as it increases risks of chronic diseases such as coronary heart disease and several types of cancer, with associated losses of quality of life and life-years [1,2]. Moreover, excessive alcohol consumption is associated with (intentional and unintentional) injuries [2,3]. In Europe, the average alcohol consumption is about 11–13 litres of pure alcohol per adult per year [3,4]. Currently about 14% of Dutch men aged 12 and over drink more than three alcoholic consumptions per day and about 10% of Dutch women aged 12 and over drink more than two alcoholic consumptions per day [5]. As a consequence of these drinking patterns, almost 7% of the burden of disease in Western Europe is alcohol related [1,2]. In the Netherlands, about 1% of the mortality, 4.5% of the burden of disease and 0.6% of the total health-care costs in 2003 can be attributed to chronic diseases caused by alcohol consumption [5].

Alcohol control in its various guises potentially decreases the burden of disease substantially and thus is an important instrument for health-care policy. Alcohol policies include measures to reduce the availability of alcohol, alcohol taxation, restricting the sale of alcohol, regulating the drinking context, restrictions on alcohol marketing, drink-driving counter-measures, education and persuasion, and early intervention and treatment services [6]. Alcohol tax increase is a public policy tool that traditionally falls outside the scope of health policy. The reason for this might be that alcohol taxes usually are controlled by ministries of finance and that tax revenues are not part of the health-care budget. A notable exception in this respect is Thailand where excise taxes on alcohol and tobacco are used to fund major health-care programmes [7]. Still, from a public health perspective alcohol taxes are an important instrument, as they are known to be able to decrease alcohol consumption [6,8]. Tax increases have been shown to decrease, for instance, cirrhosis mortality and drink-driving deaths [2].

Since in most Western countries retail prices of alcohol are heavily influenced by alcohol excise taxes [3], an increase in alcohol taxes normally leads to an increase in prices of alcohol, which in turn leads to a decrease in the demand for alcohol. To measure the effect of price increases on alcohol consumption, economists estimate the price elasticity that indicates how much the consumption of a certain good changes if its price is changed. Clements et al [9] report price elasticity figures for seven countries (Finland, Sweden, Norway, UK, Canada, Australia and New Zealand), covering the period from the mid-1950s to the mid-1980s [9]. They find a price elasticity of -0.35 for beer, -0.68 for wine and -0.98 for spirits. These price elasticity figures imply that if, for instance, beer prices increase by 10%, beer consumption decreases by 3.5%.

So, increasing alcohol taxes may be an effective strategy to control alcohol consumption, but is it also cost-effective? The information needed to answer this question can be provided by an economic evaluation. An economic evaluation is a comparative analysis of the costs and effects of two or more interventions [10]. The outcome of an economic evaluation is expressed as a ratio of incremental costs to incremental effects: the incremental cost-effectiveness ratio (ICER). To our knowledge, the only previous study on the cost-effectiveness of alcohol taxation was conducted within the World Health Organization (WHO)-CHOICE project [11,12]. They found that alcohol control policies, particularly tax increases on alcohol, are cost-effective relative to other health interventions. However, in their estimates of the cost-effectiveness of alcohol taxes, they only took into account health effects derived from heavy drinkers and ignored the effects of taxation on the health of moderate drinkers.

In this paper, we estimate the cost-effectiveness of an increase in alcohol tax using a dynamic model for the entire Dutch population from a health-care perspective, focusing on health benefits and health-care costs in alcohol users themselves. This implies that we will not take into account the external effects of alcohol prevention policies (for example, through a reduction of harm done to others through violence). Also, since knowledge is only available on the effects of price increases on average consumption of alcohol, we were unable to take into account the effects on specific subgroups with deviant drinking patterns (for example, binge drinking on 1 or 2 days a week and abstinence on the remaining days) and on alcohol dependency.

## Methods

### The National Institute for Public Health and the Environment chronic disease model

To extrapolate from decreased alcohol consumption due to tax increases to effects on health-care costs, life-years gained and quality-adjusted life-years (QALYs) gained, the Dutch National Institute for Public Health and the Environment chronic disease model (CDM) was used [13-17]. The CDM is a tool to describe the morbidity and mortality effects of risk factors for chronic diseases, such as smoking and overweight, and has been used for projections of risk factor and disease prevalence, estimates of health-adjusted life expectancy and cost-effectiveness analysis. This population model describes the life course of cohorts in terms of changes between risk factor classes and changes between disease states over time. It allows for co-morbidity and includes data on the most important chronic diseases and their risk factors. Risk factors and diseases are linked through relative risks on disease incidence. The model relates the risk factor alcohol use to the following diseases: coronary heart disease, stroke, oesophageal cancer, breast cancer, oral cavity cancer and laryngeal cancer. Alcohol classes distinguished in the CDM are for women: abstinence (no alcohol consumption), moderate alcohol consumption (fewer than two standard drink units per day), excessive alcohol consumption (between two and four standard drink units per day) and dangerous alcohol consumption (more than four standard drink units per day), and for men: abstinence (no alcohol consumption), moderate alcohol consumption (fewer than four standard drink units per day), excessive alcohol consumption (between four and six standard drink units per day) and dangerous alcohol consumption (more than six standard drink units per day) [18].

Table 1 displays the relative risks for diseases related to alcohol consumption and all-cause mortality employed in the CDM, which are all derived from a meta-analysis [18]. From Table 1, it can be seen that alcohol consumption has a positive influence on coronary heart disease and stroke (with the exception of dangerous drinking), but a negative influence on the incidence of several types of cancers. Dangerous levels of alcohol consumption are also negatively related to stroke incidence. To capture the influence of increased mortality caused by alcohol through causes of death not explicitly modelled in the CDM, a relative risk of other causes of death is employed in the CDM [14]. In this way, the influence of alcohol on mortality caused by injuries (for example, traffic accidents) and non-CDM diseases (for example, liver cirrhosis) is taken into account.

**Table 1:** Relative risks^1 ^of four different categories of alcohol consumption on disease incidence and all-cause mortality, both for men (upper row) and women (lower row)

	**Abstinence**	**Moderate**^2^	**Excessive**^2^	**Dangerous**^2^
Coronary heart disease	1	0.82	0.84	0.88
	1	0.82	0.84	0.88
Stroke	1	0.60	0.92	1.79
	1	0.58	0.48	7.96
Oesophageal cancer	1	1.80	2.37	4.26
	1	1.80	2.37	4.26
Breast cancer	1	1	1	1
	1	1.09	1.31	1.68
Laryngeal cancer	1	1.83	3.90	4.93
	1	1.83	3.90	4.93
Oral cavity cancer	1	1.45	1.85	5.39
	1	1.45	1.85	5.39
All-cause mortality	1	0.91	1.15	1.35
	1	0.96	1.35	1.49

^1^For 95% confidence intervals for all relative risks, see Holman et al [18].

^2^Definitions of alcohol consumption categories:

moderate: fewer than two standard drinks (< 20 g) per day for women, and fewer than four standard drinks (< 40 g) per day for men;

excessive: 2–4 standard drinks (20–40 g) per day for women, and 4–6 standard drinks (40–60 g) per day for men;

dangerous: more than four standard drinks (> 40 g) per day for women, and more than six standard drinks (> 60 g) per day for men.

Table 1 shows the challenge and difficulties of alcohol prevention policies, as moderate and even excessive consumption of alcohol not only has negative public health effects, but positive effects as well. It should be noted that oesophageal, laryngeal and oral cavity cancers have a low incidence compared with cardiovascular disease, stroke and breast cancer. On balance, excessive and dangerous levels of alcohol consumption have been shown to have an elevated mortality risk [11,19].

### Scenarios

To evaluate the long-term effects of alcohol tax increases, the following two intervention scenarios were compared with the current practice scenario (excise taxes as implemented in 2007 in the Netherlands).

0 Current practice: in the Netherlands the taxes on alcohol are 8 cents per bottle of beer (0.3 litre), 44 cents per bottle of wine (0.75 litre), and 371 cents per bottle of spirit (0.7 litre) [20].

1 'Dutch scenario': health effects and cost-effectiveness of the tax increase as currently planned for 2009 are estimated. The government has planned a tax increase on beer of 2.7 cents per bottle of beer (0.3 litre) [21]. As currently planned, excise taxes for spirits will remain unchanged compared with the current practice scenario. It is assumed that producers fully pass on the tax increase to consumers.

2 'Swedish scenario': health effects and cost-effectiveness of a tax increase are estimated, assuming that tax levels are increased to the same level as in Sweden, which has one of the highest alcohol taxes in the EU. This would imply that taxes on beer would be increased by €0.18 per bottle of beer, wine by €1.34 per 75 cl and spirits by €9.51 per 70 cl [20]. In this scenario it is also assumed that producers fully pass on the tax increase to consumers.

In the current practice scenario, we made projections with the CDM of QALYs and health-care costs using the distribution of alcohol consumption. This distribution was estimated using data from the annual General Public Health and Lifestyle Survey (Dutch initials: POLS) from Statistics Netherlands. These were then compared with the intervention scenarios. We estimated alcohol consumption distribution, which had been changed due to the alcohol tax increases, using the price elasticity estimates published by Clements et al [9]. This was carried out by first subtracting the average decrease in alcohol consumption due to the tax increase for every individual on the raw POLS data. Then, the alcohol consumption distribution was re-estimated. To calculate the average relative decrease in alcohol consumption due to absolute tax increases for the different types of alcohol, the absolute price increase had to be transformed in a relative price increase. To do this, data on the market shares and prices of beer, wine and spirits were needed. Market shares of the different types of alcohol were taken from Cnossen [3] and a range of sales prices was taken using supermarket prices as a minimum and catering industry prices as a maximum. Although there are some (administrative) costs related to alcohol tax increases, from a strict health-care perspective, intervention costs are absent.

To find cost-effectiveness ratios, yearly differences in model outcomes between intervention and current practice scenarios were discounted and added over the time horizon to find net present values for incremental life-years gained, QALYs gained and health-care costs. Future costs and effects were discounted at the Dutch standard annual percentages of 4% for costs and 1.5% for effects. The time horizon was 100 years since by then the cohorts that experienced the price increase have become extinct.

Data for cost of illness in the Netherlands for 2003 were used to estimate health-care expenditure in the different scenarios [22]. Average (age- and sex-specific) annual costs per patient having a certain disease were calculated by dividing the total annual costs by Dutch prevalence numbers for each disease in 2003. First, the annual disease costs per patient were multiplied by the projections of future prevalence numbers for each chronic disease in the model. Then, to calculate health-care costs for all 'other' diseases, the numbers of survivors were multiplied by age- and sex-specific cost profiles of 'remaining' costs. These latter are the difference between total health-care costs and the costs of the diseases incorporated explicitly in the model. Finally, these two categories of costs, one related and the other unrelated to the risk factor under study, were added to estimate annual costs. To calculate lifetime health-care costs, annual costs were added over time (see the Additional file 1 for costs per patient per year). All cost data were presented in euros at the 2003 price level.

With probabilistic sensitivity analysis (PSA), uncertainty in the input parameters is addressed and reflected in the model output (the ICER). In the PSA we explored the uncertainty around our base-case ICER estimate, associated with the uncertainty around the relative risk values [18], values of the price elasticity [9] and mean selling prices of the different types of alcohol needed to calculate the relative price increase. Table 2 summarises the assumptions in the different scenarios.

**Table 2:** Summary of assumptions and input data

	**Dutch scenario**	**Swedish scenario**
Discount rate	4% costs and 1.5% effects	4% costs and 1.5% effects
Time horizon	100 years	100 years
Target population	Current Dutch population	Current Dutch population
Price increase	Beer: 2.7 cents	Beer: 18 cents
	Wine: no increase	Wine: 134 cents
	Spirits: no increase	Spirits: 951 cents
Price elasticity beer^1^	Normal distribution	Normal distribution
	Mean: -0.35	Mean: -0.35
	Standard deviation: 0.17	Standard deviation: 0.17
Price elasticity wine^1^	Normal distribution	Normal distribution
	Mean: -0.68	Mean: -0.68
	Standard deviation: 0.54	Standard deviation: 0.54
Price elasticity spirits^1^	Normal distribution	Normal distribution
	Mean: -0.98	Mean: -0.98
	Standard deviation: 0.73	Standard deviation: 0.73
Market share different types of alcohol^2^	Beer: 44%	Beer: 44%
	Wine: 33%	Wine: 33%
	Spirits: 23%	Spirits: 23%
Mean current price of beer in €^3^	Uniform distribution 0.50–2.50	Uniform distribution 0.50–2.50
Mean current price of wine in €^3^	Uniform distribution 5.00–15.00	Uniform distribution 5.00–15.00
Mean current price of spirits in €^3^	Uniform distribution 10.00–25.00	Uniform distribution 10.00–25.00
Costs of intervention	None	None
Health-care costs	Depend on age and disease status	Depend on age and disease status
Quality-adjusted life-years	Depends on age and disease status	Depends on age and disease status

^1^Mean and standard deviation estimated using individual country estimates presented in Clements et al [9].

^2^Derived from Cnossen [3].

^3^Minimum based on supermarket prices, maximum based on catering industry.

## Results

**Figure 1:**
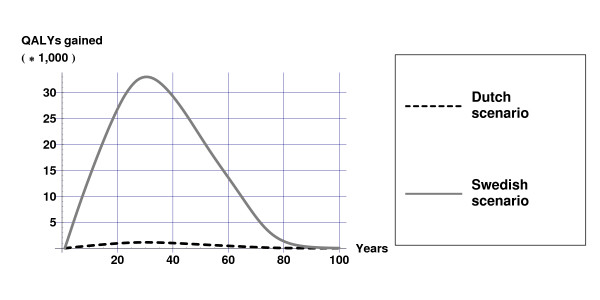
**Quality-adjusted life-years gained due to alcohol tax increases**. Quality-adjusted life-years (undiscounted) gained over time due to alcohol tax increases in both scenarios for mean values of the input parameters as displayed in table 2.

**Figure 2:**
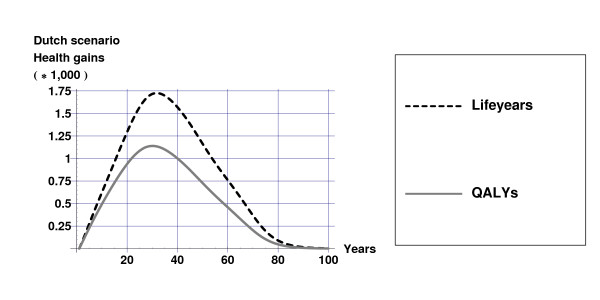
**Health gains in the Dutch scenario**. Life-years and quality-adjusted life-years (undiscounted) gained over time due to alcohol tax increases in the Dutch scenario for mean values of the input parameters as displayed in table 2.

In the Dutch scenario, alcohol consumption decreases on average by 0.3% and in the Swedish scenario, alcohol consumption decreases on average by 18.3%. The effects of these decreases in alcohol consumption on health are displayed in Figures 1 and 2 for mean values of the input parameters as displayed in Table 2. The decrease in alcohol consumption results in a decrease in the incidence of alcohol-related diseases, which causes a gain in life-years and QALYs compared with current practice. The largest effects occur some 30 years after the tax increase when the population that experienced the price increase becomes middle aged. The health gains approach zero as these cohorts become extinct. Figure 1 illustrates the large difference in health gain between the Dutch and Swedish scenarios.

**Figure 3:**
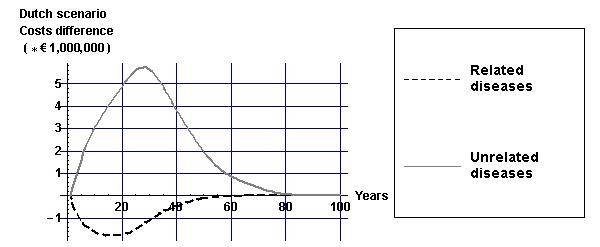
**Costs differences due to alcohol tax increases in the Dutch scenario (discounted by 4%)**. Costs differences (discounted by 4%) over time due to alcohol tax increases in the Dutch scenario for mean values of the input parameters as displayed in Table 2.

To understand the effects of alcohol tax increases on health-care costs, Figure 3 displays differences in health-care costs between the Dutch and the current practice scenarios for mean values of the input parameters as displayed in Table 2. The decrease in the incidence of diseases causally related to alcohol results in a decrease in health-care costs of those diseases. However, the gain in life-years causes an increase in the prevalence of all diseases unrelated to alcohol. From Figure 3, it can be seen that the savings in health-care costs of alcohol-related diseases are outweighed by increases in the health-care costs of diseases not related to alcohol in life-years gained.

**Figure 4:**
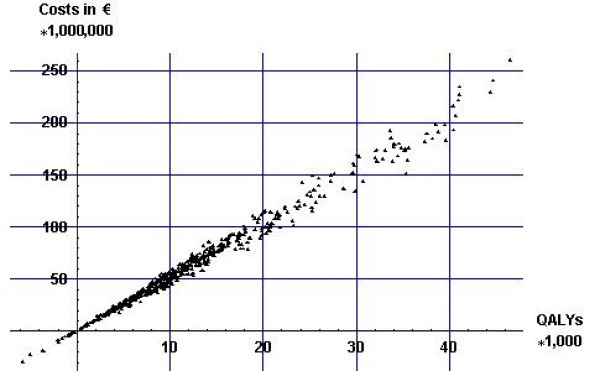
**Incremental costs and effects in the Dutch scenario**. Incremental costs and effects in the Dutch scenario for all values of the input parameters.

**Figure 5:**
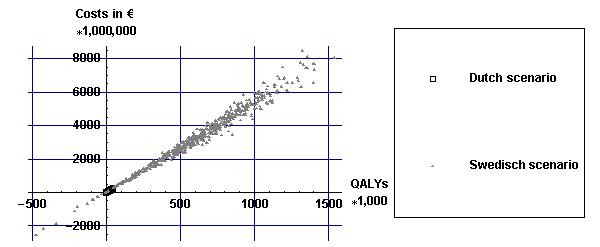
**Incremental costs and effects in the two different alcohol tax increase scenarios**. Incremental costs and effects in the two different alcohol tax increase scenarios for all values of the input parameters.

Figures 4 and 5 display cumulative differences in costs and effects (both discounted) of the two alcohol tax scenarios compared with no tax increase over a period of 100 years for all values of the input parameters. This figure illustrates the strong correlation between health gains and health-care costs. This is due to the fact that the additional health-care costs are solely the result of increases in life expectancy from a reduction in alcohol consumption. Thus, the more QALYs gained the more additional health-care costs. The health gains in the Swedish scenario are much larger than in the Dutch scenario because the price increases are much higher in the Swedish scenario.

Table 3 shows cumulative differences in health gains, cost differences and incremental cost-effectiveness ratios. Although the Swedish scenario results in many more costs and QALYs than the Dutch scenario, the costs per QALY are approximately equal. Costs per QALY are higher than costs per life-year gained in both scenarios.

**Table 3:** Incremental cost-effectiveness ratios and cumulative differences resulting from alcohol tax increases

	**Life-years gained^a^** (*1000)	**Quality-adjusted life-years gained^a^** (*1000)	**Costs differences** (* €1,000,000)^b^	**€ per life-year gained^c^**	**€ per quality-adjusted life-year gained^c^**
Dutch scenario	19 (0/57)	13 (0/39)	65 (-/191)	3500	5100
Swedish scenario	930 (-11/1909)	624 (-7/1291)	3319 (-41/6836)	3600	5300

^a^Discounted by 1.5%.

^b^Discounted by 4%

^c^Quality-adjusted life-years and life-years gained discounted by 1.5% and costs discounted by 4%.

**Figure 6:**
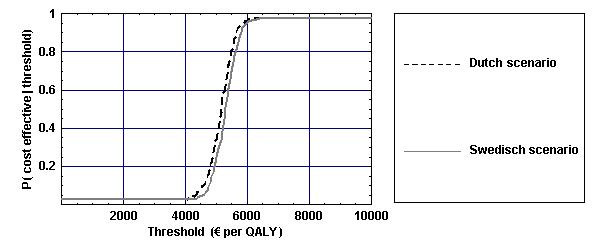
**Cost-effectiveness acceptability curves for the two different alcohol tax increase scenarios**. Figure 6 displays the cost-effectiveness acceptability curves for the two alcohol tax increase scenarios by showing the probability that an intervention is cost-effective for different values of the threshold, that is, for different monetary values placed on a QALY.

Figure 6 displays the cost-effectiveness acceptability curves (CEAC) for the alcohol tax increases. A CEAC shows the probability that an intervention is cost-effective for different values of the threshold, that is, for different monetary values placed on a QALY. What can be derived from Figure 4 is that, if QALYs are, for instance, valued at €5000, a tax increase is cost-effective with a probability of 0.5. However, if society is willing to pay €10,000 per QALY (which is below the threshold value of €20,000 per QALY that is frequently cited in the Netherlands [23,24], and far below most other estimates of the value society is willing to pay for a QALY [25]), a tax increase is cost-effective with a probability of almost one.

## Discussion

Previous research investigating the cost-effectiveness of a reduction of alcohol consumption only took into account the health effects in heavy drinkers [11,12]. However, since alcohol taxes cannot be targeted at this specific group of drinkers (the definition of heavy drinking in that study equals our categories of excessive and dangerous alcohol consumption together), health effects in the entire population, including moderate drinkers, need to be considered. Moderate drinkers even experience some disutility from a tax increase, making this policy a no Pareto improvement, but rather a Kaldor-Hicks improvement, whereby those people worse off theoretically can be compensated for by those persons that are better off with a certain policy option. Another difference with respect to the WHO-CHOICE study is how health effects were modelled. In the WHO approach, alcohol itself was modelled with a direct effect on mortality and quality of life. In our study, we modelled effects on quality of life and mortality through the effects of alcohol on alcohol-related diseases and all-cause mortality. We focused on the dynamic effects of alcohol tax increases on health effects in drinkers themselves and their associated health-care costs.

Although in theory an alcohol tax increase can be implemented by legislation alone, some administrative costs and possible costs of law enforcement to keep smuggling to a minimum have to be made to successfully implement a tax increase. These costs, as well as additional tax revenues, are usually carried by sectors outside the health-care sector. Therefore, for an alcohol tax increase, taking the health-care perspective effectively illustrated the advantages compared with interventions whose costs traditionally fall inside the health sector, such as treatment of alcohol addiction or curative treatments in a hospital setting.

Like all economic evaluation modelling studies, the calculated costs, effects and cost-effectiveness ratios are conditional on various assumptions. Below we will discuss some of the assumptions we made to estimate the cost-effectiveness of increasing alcohol taxes. First, price elasticity is the same for moderate, excessive and dangerous drinkers. If, for instance, dangerous drinkers react less to price changes than moderate drinkers [3], health effects of tax increases may be smaller than we estimated. However, there are contradictory findings on the exact price elasticity of dangerous drinkers [26,27]. Second, all tax increases were translated into price increases. We did not take into account the possibility that producers do not pass on the tax increase to consumers, resulting in a decrease in their profit margin. However, if producers only partially pass on the tax increase to consumers, this will only decrease the amount of health gains but not influence the cost-effectiveness. Third, the effects of a tax increase on alcohol consumption will be sustained in the long run. This assumption is built on studies that argue that, since alcohol consumption is addictive, the long-run price elasticity is significantly higher than the short-run elasticity. Fourth, the price elasticity is the same for high as for small price increases. This is due to the fact that estimates of price elasticity are estimated on time series with mainly small price variations over time. Again, this assumption is probably more important for the estimation of the amount of health gains rather than the cost-effectiveness ratio. Fifth, we used price elasticities based on Clements et al [9]. We preferred to use specific Dutch data, but the available estimates [28] were not specific for the different alcohol products (beer, wine, spirits). In an analysis that did not include beverages sold in restaurants, bars and hotels, Leppänen et al [28] found a high value of total elasticity for alcohol demand in the Netherlands (-1.5). However, in another analysis, they estimated the price elasticity for alcohol in the Netherlands at -0.53, which is close to the weighted average in our analysis that was based on the Clements elasticities (-0.61). Nevertheless, assuming higher values of price elasticity would result in more QALYs gained and increased health-care costs due to increases in life expectancy. For instance, modelling an elasticity of -1.5 for all alcohol types, 13,000 QALYs will be gained at costs of €65,000,000. So, while both QALYs and costs will increase, the ICER would remain almost unchanged.

In the comparative quantification of health risks study, health effects of average alcohol consumption and patterns of drinking were estimated separately [29]. In this study we have limited ourselves to the effects of a tax increase on average alcohol consumption. This was done because the CDM models average drinking and the demand elasticity estimates for alcohol also refer to average alcohol drinking. Data on the influence of patterns of drinking are less available than data on overall consumption, but evidence is accumulating that patterns of drinking affect the link between alcohol and disease and mortality [29]. For example, the same overall average volume of alcohol can be consumed in small quantities regularly with meals (for example, two drinks a day with meals) or in large quantities on a few occasions (for example, two bottles of wine on a single occasion every Friday). This also implies that we did not model the effects of a tax increase on alcohol dependence, which is a disorder in itself. The simulation model we employed did not model all diseases considered to be related to be alcohol consumption separately. Some were only modelled indirectly through an elevated mortality risk. This means that we may have underestimated the impact of alcohol consumption on quality of life and health-care costs and have overestimated the cost-effectiveness ratio. Furthermore, the relative risks employed in the CDM are not based on the most recent meta-analyses. However, the study by Holman et al [18] was the only study that included relative risk estimates for the alcohol categories employed in the CDM for both diseases and mortality, and that provided estimates of all-cause mortality. Such a category of all-cause mortality was not used in other studies on the relative risks of alcohol consumption [23]. In the simulation model we used these estimates of all-cause mortality to estimate the effects for the causes of death that are not explicitly in our model. Moreover, a recent meta-analysis of relative risks on all-cause mortality yielded similar estimates [19], and a recent study from the comparative risk assessment collaboration group used comparable estimates of relative risks for specific alcohol-related diseases [30].

In this study we have focused solely on health-care costs, ignoring broader costs and consequences of alcohol abuse to society. From a societal perspective, which is often advocated in economic evaluations [31], other costs and consequences, such as the damage due to violence and accidents induced by binge drinking, need to be considered and may be substantial [32]. Furthermore, since the price increase is not outweighed by the decrease in consumption, this also implies that tax revenues will increase if taxes are increased. It should be noted that from a societal perspective, tax revenues are transfer payments, which means that they do not increase production but simply that money flows from one place to the other. Therefore, in cost-effectiveness analyses from a societal perspective they should be omitted. However, if alcohol taxes are seen as a health policy instrument, a portion of the additional tax revenues could be added to health care [7]. Consequently, it can be argued that in this case (part of) the administrative costs and costs of law enforcement associated with tax increases should also be taken into account [13]. All in all, we expect an alcohol tax increase to be even more cost-effective when a broader societal perspective is taken.

## Conclusion

Focusing on health-care costs and health consequences for drinkers themselves, an alcohol tax increase is a cost-effective policy instrument. From a health-care perspective and taking into account health-care costs related to increased life expectancy, costs per QALY of the planned Dutch alcohol tax increase amounted on average to €5100 per QALY gained and, thus, can be considered cost-effective. A further alcohol tax increase, as is currently implemented in Sweden, can result in even more health gains and is expected to remain cost-effective because of the strong correlation between increased life expectancy and increased health-care costs.

## Abbreviations

CDM: chronic disease model; CEAC: cost-effectiveness acceptability curves; ICER: incremental cost-effectiveness ratio; POLS: General Public Health and Lifestyle Survey; PSA: probabilistic sensitivity analysis; QALY: quality-adjusted life-years; WHO: World Health Organization.

## Authors' contributions

MvdB drafted the manuscript. PHMvB carried out the analyses. RTH developed the simulation model. All authors contributed to the writing of the manuscript and approved the final manuscript.

## Competing interests

The authors declare that they have no competing interests.

## Pre-publication history

The pre-publication history for this paper can be accessed here:



## Supplementary Material

Additional file 1 **Appendix.** Disease-related costs per patient per year tables. Click here for file
