# Supplementary material for: The cost-effectiveness of increasing alcohol taxes: a modelling study
Source: BMC Med. 2008 Nov 28;6:36. doi: 10.1186/1741-7015-6-36 (PMC2637894; doi:10.1186/1741-7015-6-36)
Supplement: Additional file 1 — Appendix. Disease-related costs per patient per year tables. [file 1741-7015-6-36-S1.doc]

**Appendix**

### Disease-related costs per patient per year

**Men**

| **Age** | **Costs per patient per year in €** | | | | | | | **Costs per person per year in €** |
| --- | --- | --- | --- | --- | --- | --- | --- | --- |
|  | AMI **<Author: please give full** **name>** | AP**<Author: please give full name>** | Cerebrovascular accident | Oesophageal  cancer | Breast cancer | Laryngeal  cancer | Oral cavity cancer | Other diseases |
| 0-4 | 1100 | 2500 | 3940 | 26,300 | 670 | 2600 | 6800 | 3487.54 |
| 5-9 | 1100 | 2500 | 3940 | 26,300 | 670 | 2600 | 6800 | 1331.85 |
| 10-14 | 1100 | 2500 | 3940 | 26,300 | 670 | 2600 | 6800 | 1276.06 |
| 15-19 | 1100 | 2500 | 3940 | 26,300 | 670 | 2600 | 6800 | 1287.63 |
| 20-24 | 1100 | 2500 | 3940 | 26,300 | 670 | 2600 | 6800 | 1620.77 |
| 25-29 | 1100 | 2500 | 3940 | 26,300 | 670 | 2600 | 6800 | 1748.4 |
| 30-34 | 1100 | 2500 | 3940 | 26,300 | 670 | 2600 | 6800 | 1924.55 |
| 35-39 | 1100 | 2500 | 3940 | 26,300 | 670 | 2600 | 6800 | 2051.18 |
| 40-44 | 1100 | 2500 | 3940 | 26,300 | 670 | 2600 | 6800 | 2281.47 |
| 45-49 | 1100 | 2500 | 3940 | 26,300 | 670 | 2600 | 6800 | 2504.04 |
| 50-54 | 1100 | 2500 | 3940 | 26,300 | 670 | 2600 | 6800 | 2729.39 |
| 55-59 | 1100 | 2500 | 3940 | 26,300 | 670 | 2600 | 6800 | 3078.4 |
| 60-64 | 1100 | 2500 | 3940 | 26,300 | 670 | 2600 | 6800 | 3436.98 |
| 65-69 | 1100 | 2500 | 5120 | 26,300 | 670 | 2600 | 6800 | 4453.4 |
| 70-74 | 1100 | 2500 | 6440 | 26,300 | 670 | 2600 | 6800 | 6036.03 |
| 75-79 | 1100 | 2500 | 8100 | 26,300 | 670 | 2600 | 6800 | 8318.87 |
| 80-84 | 1100 | 2500 | 10,110 | 26,300 | 670 | 2600 | 6800 | 12,418.1 |
| 85+ | 1100 | 2500 | 12,460 | 26,300 | 670 | 2600 | 6800 | 21,779.3 |

**Women**

| **Age** | **Costs per patient per year in €** | | | | | | | **Costs per person per year in €** |
| --- | --- | --- | --- | --- | --- | --- | --- | --- |
|  | AMI**<Author: please give full** **name>** | AP**<Author: please give full** **name>** | Cerebrovascular accident | Oesophageal  cancer | Breast cancer | Laryngeal  cancer | Oral cavity cancer | Other diseases |
| 0-4 | 1100 | 2500 | 3940 | 26,300 | 1780 | 2600 | 6800 | 3125.95 |
| 5-9 | 1100 | 2500 | 3940 | 26,300 | 1780 | 2600 | 6800 | 1051. |
| 10-14 | 1100 | 2500 | 3940 | 26,300 | 1780 | 2600 | 6800 | 1096.78 |
| 15-19 | 1100 | 2500 | 3940 | 26,300 | 1780 | 2600 | 6800 | 1470.77 |
| 20-24 | 1100 | 2500 | 3940 | 26,300 | 1780 | 2600 | 6800 | 2105.06 |
| 25-29 | 1100 | 2500 | 3940 | 26,300 | 1780 | 2600 | 6800 | 2603.17 |
| 30-34 | 1100 | 2500 | 3940 | 26,300 | 1780 | 2600 | 6800 | 2980.75 |
| 35-39 | 1100 | 2500 | 3940 | 26,300 | 1780 | 2600 | 6800 | 2793.95 |
| 40-44 | 1100 | 2500 | 3940 | 26,300 | 1780 | 2600 | 6800 | 2676.01 |
| 45-49 | 1100 | 2500 | 3940 | 26,300 | 1780 | 2600 | 6800 | 2924.28 |
| 50-54 | 1100 | 2500 | 3940 | 26,300 | 1780 | 2600 | 6800 | 3177.42 |
| 55-59 | 1100 | 2500 | 3940 | 26,300 | 1780 | 2600 | 6800 | 3378.36 |
| 60-64 | 1100 | 2500 | 3940 | 26,300 | 1780 | 2600 | 6800 | 3748.1 |
| 65-69 | 1100 | 2500 | 5340 | 26,300 | 1780 | 2600 | 6800 | 4638.49 |
| 70-74 | 1100 | 2500 | 7640 | 26,300 | 1780 | 2600 | 6800 | 6427.6 |
| 75-79 | 1100 | 2500 | 11,090 | 26,300 | 1780 | 2600 | 6800 | 9558.93 |
| 80-84 | 1100 | 2500 | 15,710 | 26,300 | 1780 | 2600 | 6800 | 15,423. |
| 85+ | 1100 | 2500 | 21,480 | 26,300 | 1780 | 2600 | 6800 | 28,061. |
